# Three Transcription Activators of ABA Signaling Positively Regulate Suberin Monomer Synthesis by Activating Cytochrome P450 *CYP86A1* in Kiwifruit

**DOI:** 10.3389/fpls.2019.01650

**Published:** 2020-01-10

**Authors:** Xiaopeng Wei, Linchun Mao, Wenjing Lu, Xiaobo Wei, Xueyuan Han, Weiliang Guan, Yajie Yang, Meng Zha, Changjie Xu, Zisheng Luo

**Affiliations:** ^1^ College of Biosystems Engineering and Food Science, Zhejiang Key Laboratory of Agro-Food Processing, Key Laboratory of Agro-Products Postharvest Handling of Ministry of Agriculture and Rural Affairs, Zhejiang University, Hangzhou, China; ^2^ Ningbo Research Institute, Zhejiang University, Ningbo, China; ^3^ Institute of Food Science, Zhejiang Academy of Agricultural Sciences, Hangzhou, China; ^4^ School of Life Sciences, Shaoxing University, Shaoxing, China; ^5^ Zhejiang Provincial Key Laboratory of Horticultural Plant Integrative Biology, Zijingang Campus, Zhejiang University, Hangzhou, China

**Keywords:** kiwifruit, abscisic acid, suberin, transcriptional regulation, *N. benthamiana*

## Abstract

Wound attack stimulates accumulation of abscisic acid (ABA) that activates a number of genes associated with wound suberization of plants. Cytochrome P450 fatty acid ω-hydroxylase CYP86A1 catalyzes ω-hydroxylation of fatty acids to form the ω-functionalized monomers that play a pivotal role in suberin synthesis. However, the transcriptional regulation of ABA signaling on *AchnCYP86A1* has not been characterized in kiwifruit. In this study, *AchnCYP86A1*, a kiwifruit homolog of *Arabidopsis AtCYP86A1*, was isolated. *AchnCYP86A1*-overexpressed *N. benthamiana* leaves displayed that the AchnCYP86A1 functioned as a fatty acid ω-hydroxylase associated with synthesis of suberin monomer. The regulatory function of three transcription factors (TFs, including AchnMYC2, AchnMYB41 and AchnMYB107) on *AchnCYP86A1* was identified. All the three TFs were localized in nucleus and could individually interact with *AchnCYP86A1* promoter to activate gene expression in yeast one-hybrid and dual-luciferase assays. The findings were further demonstrated in transient overexpressed *N. benthamiana*, in which all TFs notably elevated the expression of aliphatic synthesis genes including *CYP86A1* and the accumulation of ω-hydroxyacids, α, ω-diacids, fatty acids and primary alcohols. Moreover, exogenous ABA induced the expression of *AchnMYC2*, *AchnMYB41* and *AchnMYB107* that promoted *AchnCYP86A1* involving in suberin monomer formation. Contrary to the inductive effects of ABA, however, fluridone (an inhibitor of ABA biosynthesis) inhibited the three TFs expression and suberin monomer formation. These results indicate that AchnMYC2, AchnMYB41 and AchnMYB107 positively regulate suberin monomer synthesis by activating *AchnCYP86A1* promoter in response to ABA.

## Introduction

Plants have specialized cutin and suberin that deposit in the cell walls to protect them from environmental stress, such as drought and wound pathogen attack (Pollard et al., 2008). Cutin covers all aerial parts of the plant in a primary developmental stage, whereas suberin deposition is more variable including bark tissue, fruit skin, root, seed and tuber (Sieber et al., 2000; Ranathunge et al., 2011). Furthermore, suberization is one of the hallmarks of wound damage and is well known to occur in cases of abiotic and biotic stress conditions. Chemically, suberin is a complex lipid polymer consisting of aliphatics and aromatics (Graça et al., 2015; Vishwanath et al., 2015). The aliphatics are supposed to be the main reason for the physiological important water-sealing and fungal-resisting properties of suberin (Lulai and Corsini, 1998). Aliphatics have been largely analyzed in *Arabidopsis*, potato periderm, tomato fruit and kiwifruit (Vishwanath et al., 2015; Tao et al., 2016; Han et al., 2017). Monomers released by transesterification from suberin are a mixture of ω-hydroxyacids, α, ω-diacids, fatty acids and primary alcohols with chain lengths ranging from C16 to C24, together with glycerol, and small amounts of ferulic acid (Molina et al., 2006; Jin et al., 2018). Aliphatics formation requires two main metabolic pathways including the elongation of long-chain (C16-C18) fatty acid precursors to very long-chain fatty acids (VLCFA, C20–C24), and ω-oxygenation of fatty acids to ω-hydroxyacids and α, ω-diacids (Höfer et al., 2008; Compagnon et al., 2009). The ω-functionalized suberin monomers, ω-hydroxyacids and α, ω-diacids, play a critical role in suberin assemble. The α, ω-diacids are esterified with glycerol to form the glycerol-α, ω-diacid-glycerol unit, the basic three-dimensional structure of suberin (Graça and Pereira, 2000; Graça and Pereira, 2015; Jin et al., 2018). Moreover, ω-hydroxyacids act as substrates for feruloyl transferase and glycerol 3-phosphate acyltransferase esterifying with ferulic acid and glycerol, respectively, to cross-link aliphatics and aromatics (Beisson et al., 2007; Gou et al., 2009; Molina et al., 2009; Serra et al., 2010; Yang et al., 2012). In plants ω-hydroxylation of fatty acids can be performed by cytochrome P450 monooxygenases, which is initially demonstrated in biochemical studied from *Vicia sativa* and *Pisum* (Soliday and Kolattukudy, 1977; Benveniste et al., 1982; Pinot et al., 1993). Subsequently, the AtCYP86A1 is isolated from *Arabidopsis* and found to catalyze the ω-hydroxylation of fatty acids in microsomal preparations from yeast (Benveniste et al., 1998). Mutants of *AtCYP86A1* and silencing of *StCYP86A33*, a potato homolog of *AtCYP86A1*, have been demonstrated that the CYP86A1 is mainly responsible for production of ω-hydroxyacids in suberin synthesis (Höfer et al., 2008; Serra et al., 2009). Based on these investigations, the CYP86A1 seems to be a strong candidate for the enzyme implicated in the ω-hydroxylation of fatty acids in suberin synthesis.

Wound-induced suberization and development of an impervious layer at wound sites are the major wound healing processes of plants, which can be regulated by a number of endogenous and exogenous factors (Boher et al., 2013; Fugate et al., 2016; Wei et al., 2018). Major plant hormones, such as abscisic acid (ABA), jasmonic acid (JA), salicylic acid (SA) and cytokinin, have been reported to regulate suberization processes (Mitchell and Van Staden, 1983; Lulai et al., 2008; Lulai et al., 2011; Boher et al., 2013). Accumulating evidence suggests that ABA positively regulates the suberin synthesis in kiwifruit (Han et al., 2017), tomato fruit (Leide et al., 2012), potato tuber (Lulai et al., 2008) and *Arabidopsis* root (Efetova et al., 2007). Our previous studies demonstrate that ABA can promote suberin deposition, with a concomitant up-regulation of suberin synthetic genes in kiwifruit (Han et al., 2018) and tomato fruit (Tao et al., 2016). The fluridone (FLD) can effectively block the biosynthesis of ABA (Gamble and Mullet, 1986), which has been provided a reliable mean of determining the role of ABA in wound suberization processes (Lulai et al., 2008; Tao et al., 2016).

Transcriptional regulation plays a crucial role in ABA signaling pathway. Many transcription factors (TFs) have been identified in mediating ABA regulation through the *cis*-acting regulatory elements of ABA/stress-inducible genes (Agarwal and Jha, 2010). The MYC and MYB families are found in both plants and animals with diverse functions (Elena et al., 2015). Both MYC and MYB TFs involve in the ABA-dependent pathway for the up-regulation of the abiotic stress responsive genes (Abe, 2002; Agarwal and Jha, 2010). *Arabidopsis* mutants of *AtMYB107* and *AtMYB9* exhibit a notable reduction the accumulation of ω-hydroxyacids and α, ω-diacids (Lashbrooke et al., 2016; Gou et al., 2017), while *AtMYB41*-overexpressed *Arabidopsis* and *N. benthamiana* display increases of *CYP86A1* expression and the accumulation of ω-hydroxyacids and α, ω-diacids (Kosma et al., 2015). Although knowledge of MYC2 on regulating suberin synthetic genes is unclear, the AtMYC2 positively regulates the ABA-inducible gene expression under drought stress in *Arabidopsis* plants (Abe, 2002). However, the identity of TFs directly controlling the ABA-mediated *CYP86A1* has not been revealed in kiwifruit.

In this study, *AchnCYP86A1* and three TF genes including *AchnMYC2*, *AchnMYB41* and *AchnMYB107* were isolated from kiwifruit. The functional characterization of AchnCYP86A1 as a fatty acid ω-hydroxylase was demonstrated by transient overexpressing *AchnCYP86A1* in *N. benthamiana*. The transcriptional activation of AchnMYC2, AchnMYB41 and AchnMYB107 on *AchnCYP86A1* were investigated with yeast one-hybrid, dual-luciferase, and transient overexpression in *N. benthamiana*. Moreover, genes expression and the accumulation of ω-hydroxyacids and α, ω-diacids were measured for their response to exogenous ABA.

## Materials and Methods

### Plants Materials and Treatments

Kiwifruit (*Actinidia Chinensis* Planch cv. Xuxiang), free from wound injury and infection, were harvested at commercial maturity in Hangzhou, Zhejiang Provence, China. Surface sterilization and wound treatment of fruit were performed according to our previous study (Wei et al., 2018). Subsequently, the kiwifruit halves were treated with sterile water, FLD and ABA *via* vacuum infiltration as described previously (Tao et al., 2016), and were placed into a sterile incubator (HWS, Ningbo Southeast Instrument Co., China) for wound healing at 20°C and 85% RH (relative humidity). Wound tissue samples were collected into liquid nitrogen, and then stored at −80°C for further analysis. Roots, shoots, leaves, and fruits at 35, 75, 115, and 150 days after pollination were harvested from plants, rinsed with distilled water and frozen in liquid nitrogen for temporospatial expression analysis.

### RNA Extraction and cDNA Synthesis

Total RNA was extracted from samples of kiwifruit and *N. benthamiana* leaves using the cetyltrimethylammonium bromide (CTAB) method (Reid et al., 2006). RNA concentration and 260/280 nm ratios were determined with a NanoDrop 2000 (NanoDrop Technologies, Inc., USA). Total RNA was treated with a TURBO DNA-free kit (Thermo Fisher Scientific, Inc., USA) to remove the contaminating gDNA. One μg of RNA was used for cDNA synthesis with iScript™ cDNA Synthesis kit (Bio-Rad Laboratories, Inc. USA). The cDNA was diluted fivefold and 1 µl of the diluted cDNA was used as the template for qRT-PCR analysis.

### qRT-PCR Analysis

The cDNA of kiwifruit and *N. benthamiana* genes for qRT-PCR were obtained by TBLASTX analysis against the kiwifruit genome database and the SOL Genomics Network database, respectively, using *Arabidopsis* genes as query (**Supplementary Table S1**). qRT-PCR was performed in 96 well plates using Biosystems 7500 qRT-PCR system (Thermo Fisher Scientific Inc., USA). A template-free negative controls were included for each primer pair and each PCR reaction was completed in triplicate. The relative expression levels of kiwifruit and *N. benthamiana* genes were calculated by 2^−△△Ct^ method against the *Actin* and *β-Tubulin* gene, respectively. The analysis was performed with three biological replicates.

### Yeast One-Hybrid Assay

The yeast-one hybrid assays were performed using the Matchmaker Gold Yeast One-Hybrid System Kit (TaKaRa, Ohtsu, Japan). The full-length of kiwifruit *AchnCYP86A1* was obtained by TBLASTX analysis against the kiwifruit genome database using *Arabidopsis AtCYP86A1* as query, respectively. The promoter fragment of *AchnCYP86A1* was cloned into the pAbAi vector, and AchnCYP86A1-AbAi and p35-AbAi were linearized and transformed into Y1HGold to make individual bait-reporter strains. Transformants were initially screened on plates containing a selective synthetic dextrose medium lacking uracil (SD/-Ura) with 0, 50, 80, 100, 120, 150, 200, 300, 500, 800, and 1,000 ng ml^−1^ of aureobasidin A (AbA). The full-length coding sequences (CDS) of TF genes (including *AchnMYB41*, *AchnMYB107*, *AchnMYC2*, *Achn031311*, *Achn173251*, *Achn313331*, *Achn318681*, *Achn136071*, *Achn310341*, *Achn313181*, and *Achn084621*) were cloned into pGADT7 (AD) vector and transformed into the individual bait-reported strains. The transformed Y1HGold were cultured on SD medium without leucine supplemented with 120 ng ml^−1^ AbA (SD/-Leu+AbA^120^) at 28°C for 3 d. The primers are listed in **Supplementary Table S2**.

### Dual Luciferase Assay

Dual luciferase assays were performed with *N. benthamiana* according to Min et al. (2012). In brief, full-length CDS of the *AchnMYC2*, *AchnMYB41*, and *AchnMYB107* were cloned into pGreen II 0029 62-SK vector (SK), respectively, and the promoter fragment of *AchnCYP86A1* was inserted into the pGreen II 0800-LUC vector. All the constructs were individually electroporated into *Agrobacterium tumefaciens* GV3101 by freeze-thaw method. *Agrobacterium* culture mixtures harboring constructs of *AchnCYP86A1* promoter and the individual TF genes were infiltrated into *N. benthamiana* leaves using needleless syringes. FLUC (Firefly luciferase) and RLUC (Renilla luciferase) activities were determined 72 h after infiltration using the Dual-Luciferase Reporter Assay System (E710, Promega, USA) with Modulus Luminometers (GloMax96, Promega, USA). Luciferase activity was analyzed in three independent experiments. The primers are listed in **Supplementary Table S2**.

### Plasmid Construction and Transient Overexpression

The full-length CDS of *AchnCYP86A1*, *AchnMYC2*, *AchnMYB41*, and *AchnMYB107* were individually cloned into plant binary vector pBI121 replacing the GUS gene, behind the CaMV 35S promoter. The recombinant plasmids were transformed into *Agrobacterium* cells (EHA105) and grown to saturation in liquid yeast extract peptone. After centrifugation, the pellet was re-suspended in the infection solution (10 mM MES, 10 mM MgCl_2_ and 150 mM acetosyringone). Five weeks-old *N. benthamiana* leaves were used for infiltration, and the *pBI121* empty vector (*pBI121* Ev) infiltrated into *N. benthamiana* leaves was set as control. Leaves were harvested for qRT-PCR and suberin monomer analysis six days after infiltration. The primers are listed in **Supplementary Table S2**.

### Subcellular Localization Analysis

Subcellular localization of AchnMYC2, AchnMYB41 and AchnMYB107 according to Voinnet et al. (2003). The CDS of the three TF genes without termination codons were independently inserted into pBI221-EGFP and transient overexpressed under the control of the CaMV 35S promoter. The fusion construct 35S:MYC2-GFP, 35S:MYB41-GFP, 35S:MYB107-GFP, and control vector pBI221-EGFP were transformed into *Agrobacterium* cells (EHA105). *Agrobacteria tumefaciens* lines harboring the vectors were independently infiltrated into *N. benthamiana* leaves. The green fluorescent proteins in leaves were observed after 3 d infiltration under a confocal microscope (TCS SP8, Leica) with excitation wavelengths at 488 nm, respectively. The primers are listed in **Supplementary Table S2**.

### Suberin Depolymerization and Monomer Analysis

Soluble lipid fraction was extracted as described by Legay et al. (2016). The dry residue was depolymerized using acid-catalyzed methanolysis (Debolt et al., 2009). In brief, the samples and internal standards (methyl heptadecanoate and ω-pentadecalactone) were adding sulfuric acid/methanol (1:20, v/v) in glass vials, and immediately incubated at 85°C for 3 h. Suberin monomers were extracted by adding two volumes of dichloromethane and one volume of 0.9% (w/v) NaCl. After aqueous washing, the organic phase was dried over anhydrous sodium sulfate and evaporated under nitrogen gas. The residues were derivatized by adding 100 µl of pyridine and 100 µl of BSTFA (*N*, *O*-bis (trimethylsilyl)-trifluoroacetamide) at 70°C for 40 min. The samples were dried under nitrogen gas and dissolved by 500 µl dichloromethane. The 20 µl samples were analyzed on Agilent Technologies 7890B-5977A Gas Chromatograph-Mass Spectrometer Detector (GC–MSD) system (Han et al., 2017). The analysis was performed with three biological replicates. The suberin monomers were calculated using the relative peak areas to internal standard. ω-pentadecalactone was used as internal standard for quantification of hydroxylated monomers including ω-hydroxyacids and primary alcohols, while methyl heptadecanoate was used as internal standard for determining fatty acid and α, ω-diacids.

## Results

### Gene Isolation and Analysis

**Figure 1:**
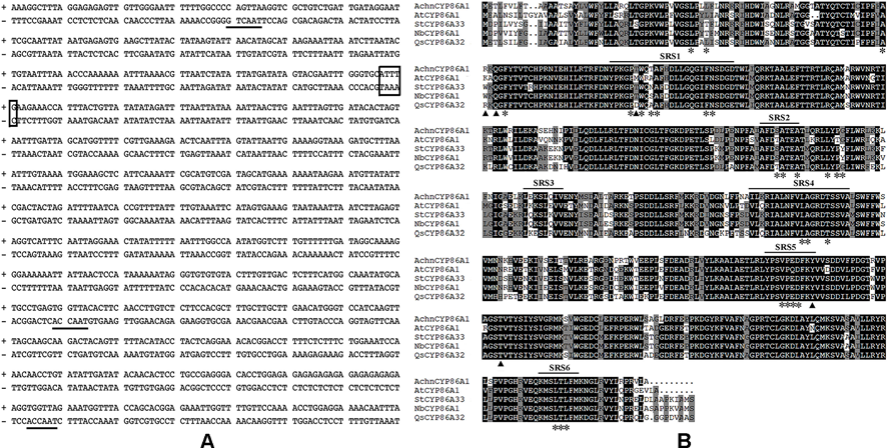
Sequence analysis of AchnCYP86A1. **(A)** The cis-acting regulatory elements of *AchnCYP86A1* promoter. Solid lines and box indicate MYB and MYC recognition elements, respectively. **(B)** Amino acid sequences of *A. chinensis* AchnCYP86A1, *A. thaliana* AtCYP86A1, *S. tuberosum* StCYP86A33, *N. benthamiana* NbCYP86A1 and *Q. suber* QsCYP86A32 were aligned using ClustalX.

The fragment (1,339 bp) of *AchnCYP86A1* promoter was obtained from kiwifruit DNA. *Cis*-acting regulatory elements of Achn*CYP86A1* promoter were analyzed using the Plant CARE database (**Figure 1A**). The promoter contained one MYC recognition element (box), and three MYB recognition elements (underlined), which could be bound by MYC and MYB proteins. At amino acid level, AchnCYP86A1 processed high similarity to *Arabidopsis* AtCYP86A1 (77%), potato StCYP86A33 (78%) and tobacco NbCYP86A1 (79%). Sequence analysis revealed that AchnCYP86A1 contained six substrate recognition sites (SRS1‒SRS6, **Figure 1B**), and the residues predicted to contact to oleic acid (asterisks). In addition, they also had the residues (arrows) likely to make hydrogen bond contact with the substrate carboxyl (Rupasinghe et al., 2010). Phylogenetic tree showed that AchnCYP86A1 was closely grouped together with StCYP86A33, AtCYP86A1 and NbCYP86A1 (**Figure S1A**), suggesting that AchnCYP86A1 was homologous to AtCYP86A1 and StCYP86A33 that could catalyze the ω-hydroxylation of fatty acids to ω-hydroxyacids in suberin synthesis (Höfer et al., 2008; Serra et al., 2009).

Based on the phylogenetic tree (**Figures S1B** and **S2**), there were many AchnMYC and AchnMYB TFs that closely grouped together with AtMYC2, AtMYB41 and AtMYB107. Firstly, yeast one-hybrid assays were used to screening the potential interacting TFs, using the *AchnCYP86A1* promoter as bait (**Figure S4**). Among six AchnMYB TFs, including AchnMYB41, AchnMYB107, Achn031311, Achn173251, Achn313331, and Achn318681, only AchnMYB41 and AchnMYB107 could individually bind to *AchnCYP86A1* promoter. For AchnMYC TFs, the five candidates, including AchnMYC2, Achn136071, Achn310341, Achn313181, and Achn084621, were also screened by yeast one-hybrid assays, and only AchnMYC2 could interact with *AchnCYP86A1* promoter. Therefore, the three TFs AtMYC2, AtMYB41 and AtMYB107 were used for further functional identification.

**Figure 2:**
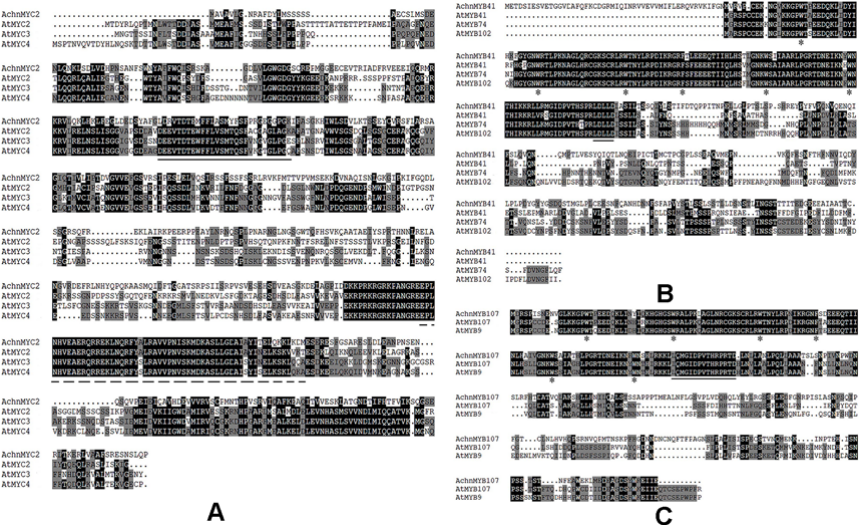
Alignment of transcription factors from kiwifruit and *Arabidopsis*. **(A**–**C)** The amino acid sequences alignment of AchnMYC2, AchnMYB41 and AchnMYB107 with homologs from Arabidopsis. The solid line indicates acidic region corresponding to activation domain of MYC2 and dash line indicates the bHLH domain. The primary structures of R2R3-MYB and the conserved motifs of MYB41 and MYB107 are indicated by arrowheads and solid line, respectively. The alignment was built using ClustalX.

Analysis of the amino acid sequence showed that AchnMYC2 shared the acidic region corresponding to activation domain of MYC2 (**Figure 2A**, solid line), and the bHLH domain functioned as the DNA-binding motif (dashed line) (Nakata et al., 2013). The AchnMYB41 and AchnMYB107 clustered with AtMYB41 and AtMYB107/AtMYB9, which belonged to R2R3 subgroup 11 and 10 proteins, respectively (**Figure S2**). Analysis of the amino acid sequences displayed that AchnMYB41 and AchnMYB107 shared the primary structures of R2R3-MYB domain (**Figures 2B**, **C**, asterisks), and the conserved motifs of their subgroups (solid line) (Kranz et al., 1998).

### Temporospatial Expression of *AchnCYP86A1*, *AchnMYC2*, *AchnMYB41*, and *AchnMYB107*

**Figure 3:**
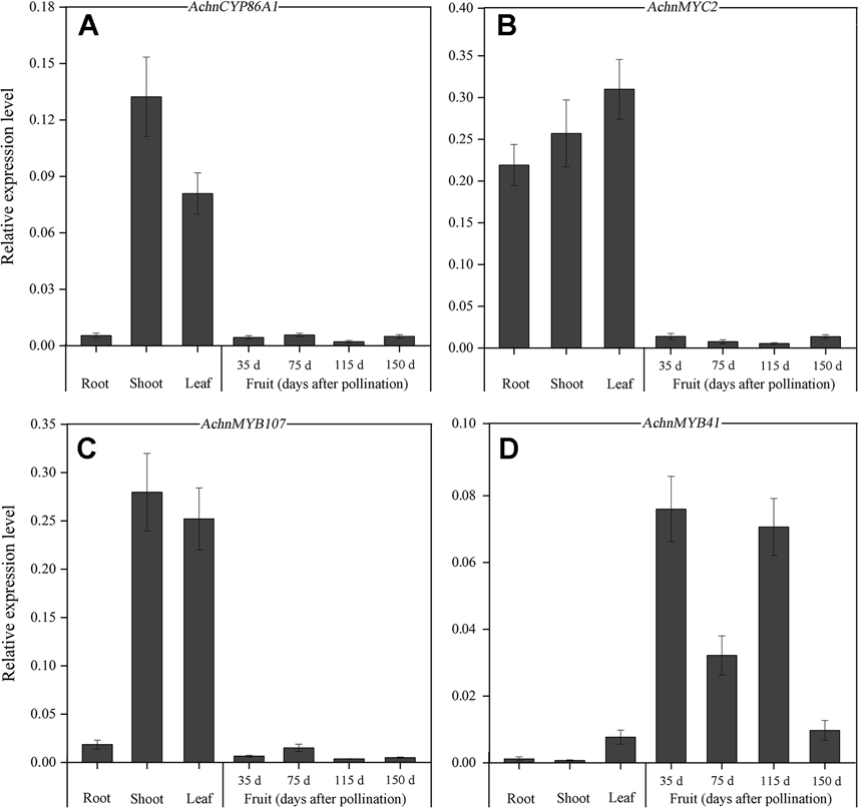
Tissue-specific expression of *AchnCYP86A1*, *AchnMYC2*, *AchnMYB41* and *AchnMYB107*. The expression of AchnCYP86A1 **(A)**, AchnMYC2 **(B)**, AchnMYB107 **(C)** and AchnMYB41 **(D)** in different kiwifruit organs and fruit. RNA was isolated from roots, shoots, leaves, and fruit at different stages of development, and reverse transcribed. Gene transcript levels were analyzed by qRT-PCR. The expression level of the genes is relative to *actin*. Error bar represents the standard deviation of three biological replicates.

The expression levels of *AchnCYP86A1*, *AchnMYC2*, *AchnMYB41* and *AchnMYB107* were assessed in various tissues using qRT-PCR (**Figure 3**). The expression of the four genes was detectable in roots, shoots, leaves, and fruit at different stages of development. Higher levels of *AchnCYP86A1* and *AchnMYB107* transcripts were detected in shoots and leaves comparing to root and fruit (**Figures 3A**–**D**), while higher levels of *AchnMYC2* transcripts could be detected in roots, shoots and leaves comparing to fruit. In contrast, *AchnMYB41* was higher expressed in fruit, especially at 35, 75 and 115 days after pollination (**Figure 3C**).

### Function of AchnCYP86A1 as a Fatty Acid ω-Hydroxylase in Suberin Monomer Synthesis

**Figure 4:**
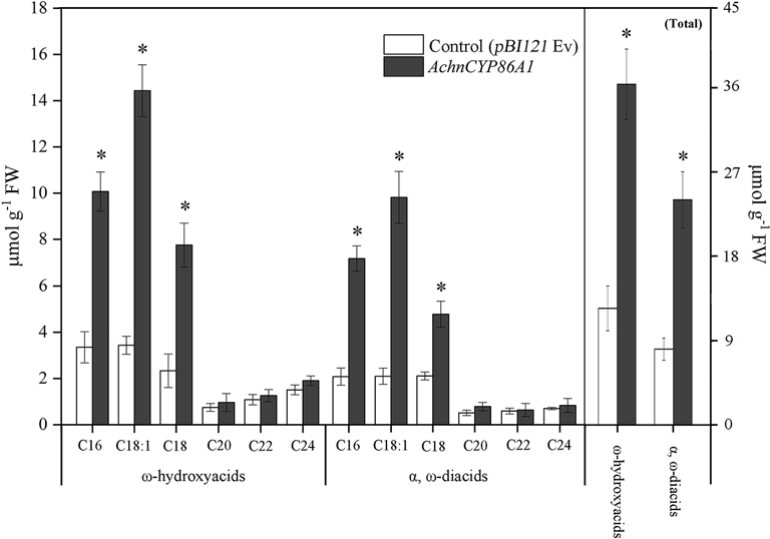
The contents of ω-hydroxyacids and α, ω-diacids, and the total amounts from control (*pBI121* Ev, empty vector) and *AchnCYP86A1*-infiltrated *N. benthamiana* leaves. Error bar represents the standard deviation of three biological replicates. Asterisk indicates significant difference (*t* test, *p value < 0.05).

In order to confirm that the AchnCYP86A1 was a fatty acid ω-hydroxylase catalyzing ω-hydroxyacids formation, the *AchnCYP86A1* was transient overexpressed in *N. benthamiana* leaves with determination of the ω-hydroxyacids and α, ω-diacids (**Figure 4**). The most pronounced increases were detected in ω-hydroxyacids with chain lengths C16–C18. In particular, the C18:1 ω-hydroxyacid increased by 4.2 of control. Similarly, the C16-C18 α, ω-diacids were also significantly induced by overexpression of *AchnCYP86A1*. However, the C20-C24 ω-hydroxyacids and α, ω-diacids showed a very minor increase. In total the amounts of ω-hydroxyacids and α, ω-diacids increased by 2.8 and 2.9-fold of control, respectively.

### Activation of AchnMYC2, AchnMYB41, and AchnMYB107 on *AchnCYP86A1* Promoter

**Figure 5:**
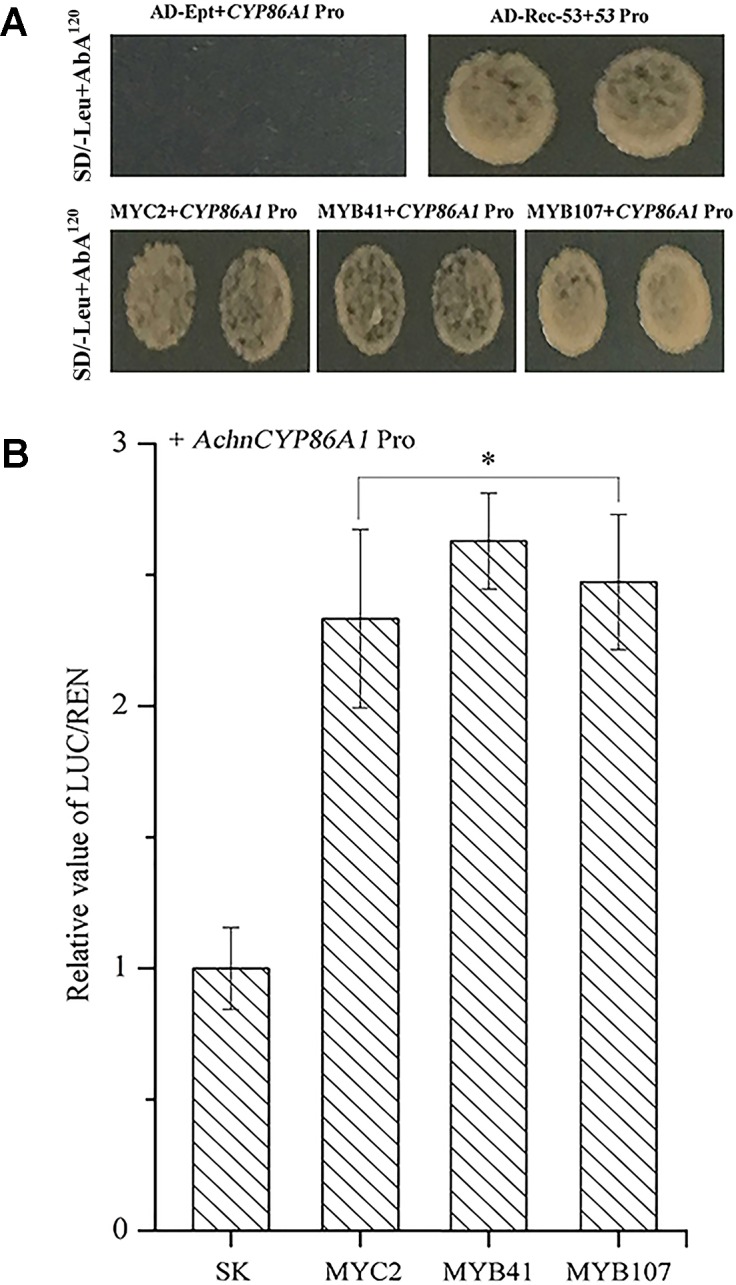
The AchnMYC2, AchnMYB41, and AchnMYB107 individually activate the *AchnCYP86A1* promoter. **(A)** Yeast one-hybrid assay of AchnMYC2, AchnMYB41 and AchnMYB107 to the *AchnCYP86A1* promoter. AD-Rec-p53 with p53-AbAi was used as a positive control, while AD-empty with AchnCYP86A1-AbAi was used as a negative control. **(B)** AchnMYC2, AchnMYB41 and AchnMYB107 activate the *AchnCYP86A1* promoter in dual-luciferase assays. The ratio of LUC/REN of the empty vector (SK) plus promoter was used as calibrator (set as 1). Pro, promoter; Ept, empty. Error bar represents standard deviation of three independent experiments. Asterisk indicates significant difference (*t* test, *p value < 0.05).

Yeast one-hybrid assays were used to investigate whether AchnMYC2, AchnMYB41 and AchnMYB107 could directly bind to *AchnCYP86A1* promoter. For auto-activation analysis, the Y1HGold harboring AchnCYP86A1-AbAi was suppressed by 120 ng ml^−1^ of AbA. The interaction test showed that expression of AchnMYC2, AchnMYB41, and AchnMYB107 independently induced the expression of the resistance reporter gene AbA driven by the *AchnCYP86A1* promoter (**Figure 5A**). The results indicated that AchnMYC2, AchnMYB41, and AchnMYB107 could directly interact with *AchnCYP86A1* promoter. The activation of the three TFs on *AchnCYP86A1* promoter were detected using dual luciferase assay in *N. benthamiana* leaves (**Figure 5B**). AchnMYC2, AchnMYB41, and AchnMYB107 noticeably activated *AchnCYP86A1* promoter, with the increasement of 2.3, 2.6, and 2.5-fold, respectively.

### Positive Regulation of *AchnMYC2*, *AchnMYB41,* and *AchnMYB107* on Suberin Synthetic Genes and Monomers Accumulation

**Figure 6:**
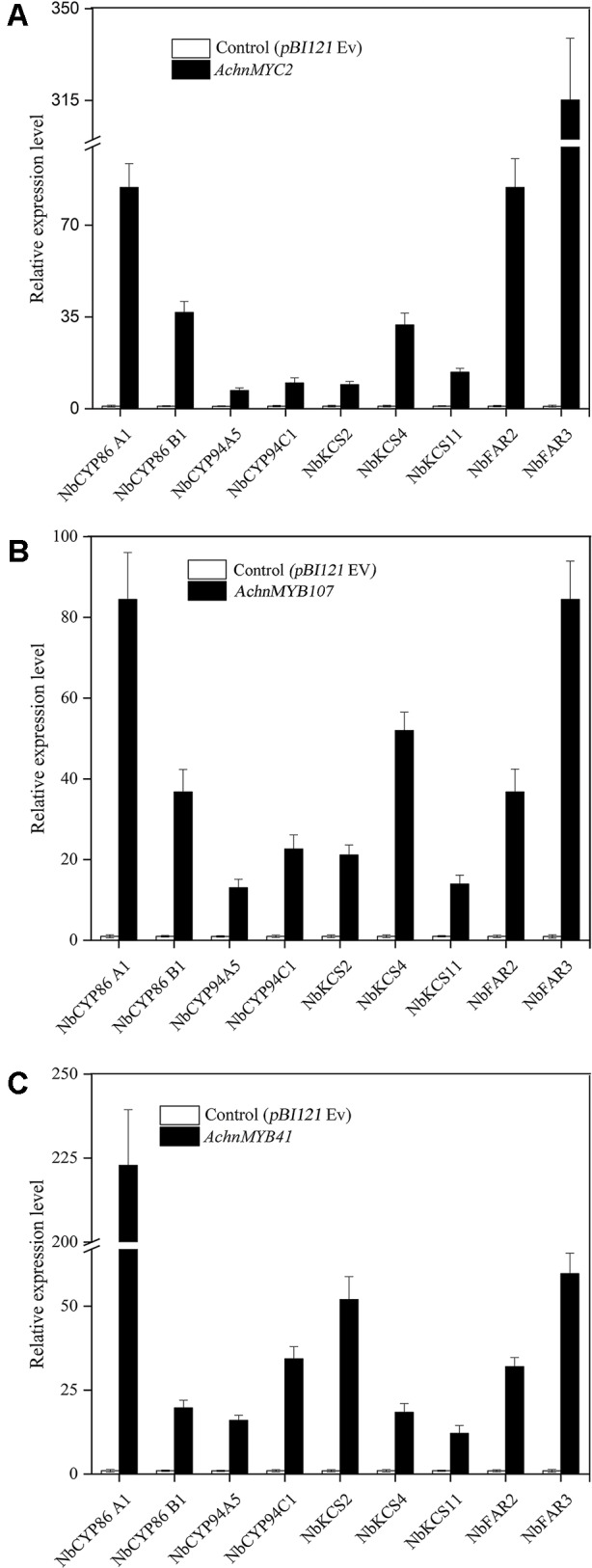
Relative expression change of suberin synthetic genes in *N. benthamiana* leaves after 6 days of *AchnMYC2* **(A)**, *AchnMYB41* **(C)** and *AchnMYB107* **(B)** overexpression against control (*pBI121* empty vector). Error bar represents the standard deviation of three biological replicates.

To further confirm the functions of *AchnMYC2, AchnMYB41*, and *AchnMYB107* on suberin synthesis, the three TF genes were separately transient overexpressed in *N. benthamiana* leaves and expression of aliphatic genes were detected (**Figure 6**), using the genes involved in glucose metabolism as negative control (**Figure S3**). Genes involved in glucose metabolism, including *SPS* (*sucrose-phosphate synthase*), *HK* (*hexokinase*), *AGP* (*ADP glucose pyrophosphorylase*) and *UGP* (*UDP glucose pyrophosphorylase*), were not significantly altered by overexpression of the TF genes (**Figure S3**), which confirmed the role of the TFs on aliphatic genes. The genes involved in aliphatics synthesis were strongly induced by overexpression of *AchnMYC2, AchnMYB41,* and *AchnMYB107*. The *N. benthamiana* homolog of *AchnCYP86A1*, *NbCYP86A1*, was significantly induced by overexpression of *AchnMYC2, AchnMYB41,* and *AchnMYB107*, with increasement of 6.4, 7.8, and 6.4-fold of control, respectively. Additionally, gene encoding a VLCFA ω-hydroxylase (NbCYP86B1) (Compagnon et al., 2009) was also significantly elevated. The AtCYP94C1 and NtCYP94A5 proteins have been reported catalyzing the oxidation of fatty acids to corresponding α, ω-diacids *in vitro* (Le Bouquin et al., 2001; Kandel et al., 2007). *NbCYP94C1* and *NbCYP94A5*, the homologs of *AtCYP94C1* and *NtCYP94A5*, were strongly induced. The 3-ketoacyl-CoA synthase (KCS) genes, *NbKCS2*, *NbKCS4* and *NbKCS11*, were significantly increased. The KCS proteins are part of the fatty acid elongation complex generating VLCFA in suberin synthesis (Vishwanath et al., 2015). Genes encoding fatty acyl-reductases (NbFAR2 and NbFAR3), which catalyze the synthesis of fatty alcohols in suberization processes (Domergue et al., 2010), were also strongly up-regulated.

**Figure 7:**
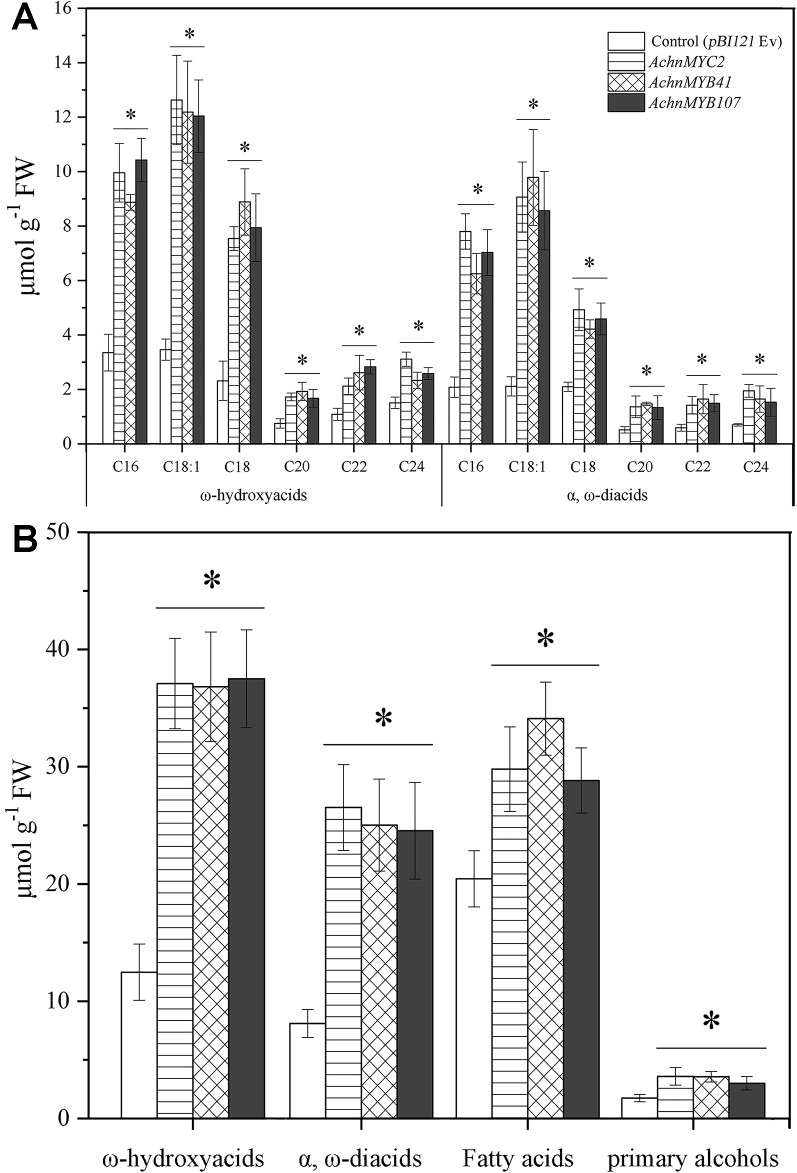
The contents of ω-hydroxyacids and α, ω-diacids from control (*pBI121* Ev, empty vector) and *AchnMYC2*, *AchnMYB41* and *AchnMYB107* infiltrated *N. benthamiana* leaves. **(A)** The amount of ω-hydroxyacids and α, ω-diacids. **(B)** The total amounts of ω-hydroxyacids, α, ω-diacids, fatty acids and primary alcohols. Error bar represents the standard deviation of three biological replicates. Asterisk indicates significant difference (*t* test, *p value < 0.05).

In chemical analysis, the ω-functionalized suberin monomers, ω-hydroxyacids, and α, ω-diacids, were significantly increased by overexpression of *AchnMYC2*, *AchnMYB41*, and *AchnMYB107* (**Figure 7A**). Significant increases of ω-hydroxyacids with chain lengths C16–C18, which were mainly produced by CYP86A1, were detected in leaves overexpression of *AchnMYC2*, *AchnMYB41*, and *AchnMYB107*. Specifically, the C18:1 ω-hydroxyacid was increased by 3.5 to 3.6-fold relative to control. The 20–24 ω-hydroxyacids mainly generated by CYP86B1 were also significantly induced by the three TF genes. The α, ω-diacids were probably produced by CYP94C1 and CYP94A5 (Le Bouquin et al., 2001; Kandel et al., 2007). In agreement with the expression of *CYP94C1* and *CYP94A5*, α, ω-diacids were significantly elevated by overexpressing the three TF genes. In addition, the amounts of fatty acids and primary alcohols, which are generated by KCS and FAR proteins, were significantly induced by overexpression of *AchnMYC2*, *AchnMYB41,* and *AchnMYB107* (**Figure 7B**).

### Subcellular Localization

**Figure 8:**
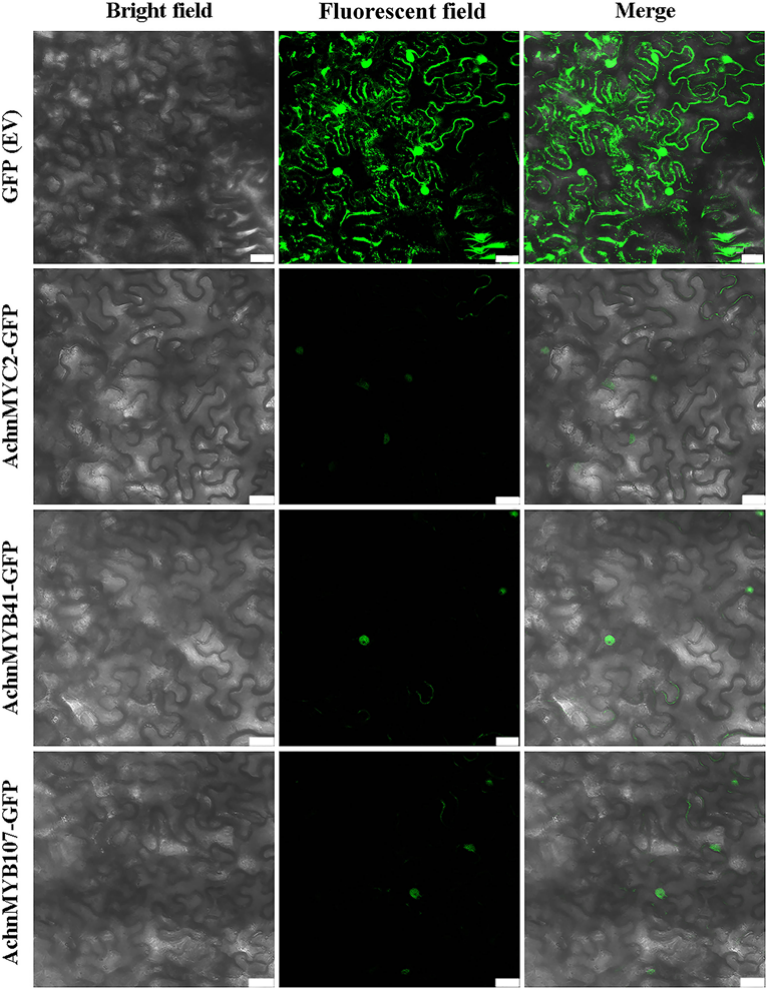
Subcellular localization of AchnMYC2, AchnMYB41 and AchnMYB107 in *N. benthamiana* leaves. The GFP empty vector is transformed into *N. benthamiana* leaves as control. Ev, empty vector. Bar, 75 μm.

Subcellular localization of AchnMYC2, AchnMYB41, and AchnMYB107 were analyzed in *N. benthamiana* leaves by GFP tagging with the GFP (Ev, empty vector) as control (**Figure 8**). The green fluorescent signals of GFP (Ev) were observed in the whole cells, while signals of AchnMYC2-GFP, AchnMYB41-GFP and AchnMYB107-GFP proteins were observed in nucleus.

### Induction of Gene Expression and Suberin Monomer Accumulation by Exogenous ABA

**Figure 9:**
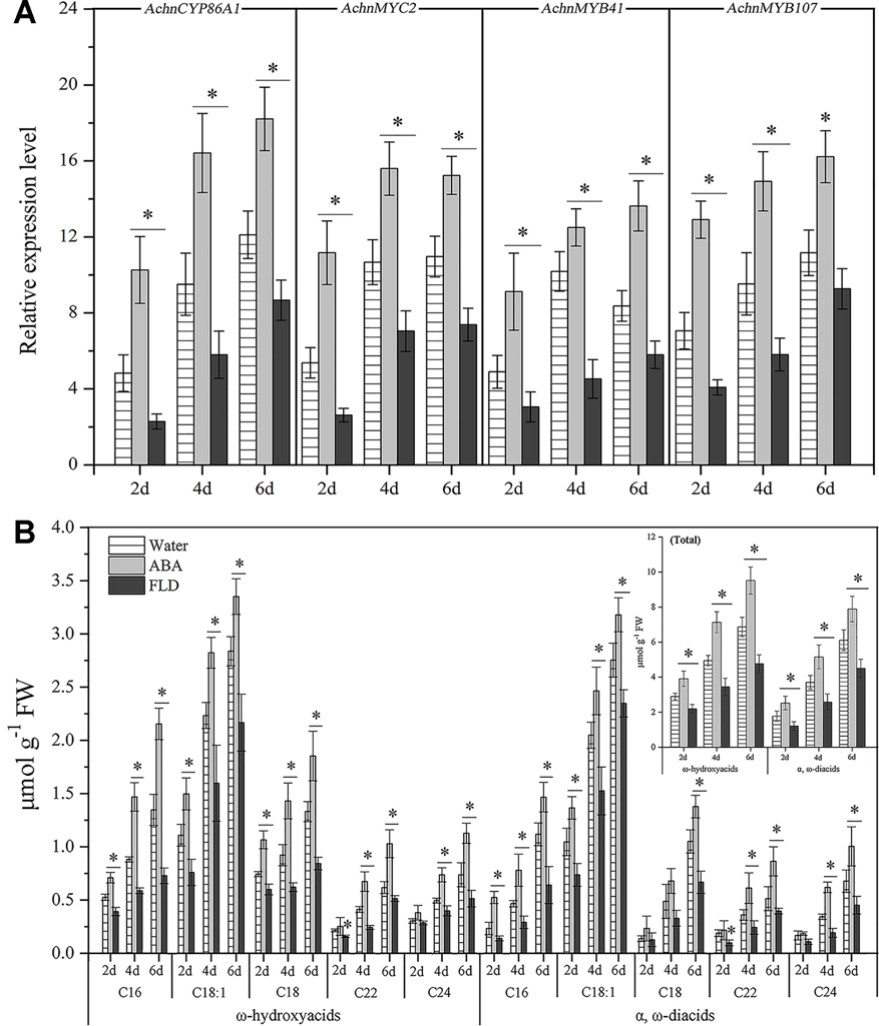
Genes expression and the content of ω-hydroxyacids and α, ω-diacids in kiwifruit wound tissues treated with water, ABA and FLD. **(A)** Expression levels of *AchnCYP86A1*, *AchnMYC2*, *AchnMYB41* and *AchnMYB107*. Relative mRNA abundance was evaluated by qRT-PCR and presented as fold change relative to the initial value upon wounding. **(B)** Accumulation of ω-hydroxyacids and α, ω-diacids. Inset graph shows total content of ω-hydroxyacids and α, ω-diacids. ABA, abscisic acid; FLD, fluridone. Error bar represents the standard deviation of three biological replicates. Asterisk indicates significant difference (*t* test, *p value < 0.05).

The expression of *AchnCYP86A1*, *AchnMYC2*, *AchnMYB41,* and *AchnMYB107* in water, ABA or FLD treated wound tissues of kiwifruit were investigated (**Figure 9A**). The *AchnCYP86A1*, *AchnMYC2*, *AchnMYB41,* and *AchnMYB107* were up-regulated by ABA treatment, while the FLD suppressed the genes expression over 6 d of wound healing. Compared with water treatment, *AchnCYP86A1* expression levels were increased by 1.5 and 0.7-fold in ABA and FLD treatments at 6 d of wound healing, respectively. Expression levels of *AchnMYC2*, *AchnMYB41*, and *AchnMYB107* in ABA treatment were also elevated up to 1.4, 1.6 and 1.5-fold of water treatment at 6 d of wound healing, while FLD significantly inhibited their expression.

Consistent with the genes expression, exogenous ABA significantly elevated the accumulation of ω-hydroxyacids and a, ω-diacids with chain lengths C16–C24, while FLD treatment strongly reduced their formation (**Figure 9B**). by 6 d of wound healing, the total content of ω-hydroxyacids in ABA treatment amounted to 9.5 µmol g^−1^, with increasement of 1.4 and 2.0-fold relative to water and FLD treated tissues, respectively. likewise, total content of a, ω-diacids in ABA treatment was 1.3 and 1.8-fold relative to water and FLD treatments, respectively (**Figure 9B**, inset).

## Discussion

Suberization is a common plant response to wounding and plays a critical role in formation of an impervious layer (Han et al., 2017; Wei et al., 2018). The aliphatics are considered as critical composition in controlling excess water loss and pathogen infection from wound tissues (Lulai and Corsini, 1998). Genes involved in aliphatics synthesis have been identified in potato, *Arabidopsis* and cork (Soler et al., 2007; Vishwanath et al., 2015). Among them, *Arabidopsis AtCYP86A1* and potato *StCYP86A33* are important for generation of ω-functionalized suberin monomers, ω-hydroxyacids and α, ω-diacids that are essential for suberin assemble (Höfer et al., 2008; Serra et al., 2009; Graça et al., 2015). The catalytic property of AtCYP86A1 *in vitro* is identified from yeast expressing *CYP86A1*, which actively metabolized fatty acids with chain lengths C12–C18 to corresponding ω-hydroxyacids, showing a preference for C16 and C18:1 fatty acids (Benveniste et al., 1998). *StCYP86A33*-silenced potato and *Arabidopsis* mutants of *AtCYP86A1* both exhibit significantly reduction of C16-C18 (specifically C18:1) ω-hydroxyacids and α, ω-diacids (Höfer et al., 2008; Serra et al., 2009). In the current study, the kiwifruit AchnCYP86A1 showed high similarity to AtCYP86A1, StCYP86A33 and NbCYP86A1 with the substrate recognition sites (SRS1‒SRS6) and the residues contacting oleic acid (**Figure 1B**). The highly conserved motifs among AchnCYP86A1, AtCYP86A1 and StCYP86A33 probably resulted in the similarity function. Pronounced increases in long-chain (C16–C18) ω-hydroxyacids and *AchnCYP86A1* expression were detected in wound tissues of kiwifruit (**Figure 9**). In *N. benthamiana* leaves, overexpression of *AchnCYP86A1* led to significantly production of long-chain ω-hydroxyacids, especially C18:1 ω-hydroxyacid. However, the very long-chain (C20–C24) ω-hydroxyacids showed minor increases (**Figure 4**). Additionally, the long-chain α, ω-diacids were also significantly increased. The long-chain α, ω-diacids accumulation could be performed in two different ways including that through dehydrogenase reactions of long-chain ω-hydroxyacids to generate the corresponding α, ω-diacids, and AchnCYP86A1 probably acted as a multifunctional ω-hydroxylase, similar to CYP94A5 and CYP94C1 catalyzing the multi-step oxidation of fatty acids to corresponding α, ω-diacids (Le Bouquin et al., 2001; Kandel et al., 2007). In kiwifruit, the expression of *AchnCYP86A1* and the accumulation of ω-hydroxyacids were highly induced by wounding (**Figure 9**). Therefore, the results demonstrate that AchnCYP86A1 functions as a fatty acid ω-hydroxylase involved in wound suberization of kiwifruit.

It is commonly recognized that MYB TFs play a regulatory role in suberin synthesis pathway. Three *Arabidopsis* MYB TFs (AtMYB9, AtMYB41, and AtMYB107) and apple MdMYB93 have been shown to positively regulate suberin synthesis. AtMYB41 and MdMYB93 work as transcriptional activators showing a correlation between gene overexpression, up-regulation of the key suberin synthetic genes (such as *CYP86A1*, *KCS*, *FAR,* and *ASFT/HHT*) and suberin monomers deposition (Kosma et al., 2015; Legay et al., 2016). *Arabidopsis* mutants of *AtMYB9* and *AtMYB107* display a notable reduction in suberin monomers and down-regulation of suberin synthetic genes (Lashbrooke et al., 2016; Gou et al., 2017). AtMYC2 functions as a transcriptional activator in ABA signaling to regulate dehydration-responsive gene (Abe, 2002). In this study, kiwifruit AchnMYB41 and AchnMYB107 shared high homology with AtMYB41 and AtMYB107/AtMYB9 on N-terminal region sharing R2R3 MYB domain, respectively (**Figures 2B**, **C**), while the AchnMYC2 shared the conserved bHLH domain of AtMYC2 (**Figure 2A**). Transient overexpression of *AchnMYB41*, *AchnMYB107* and *AchnMYC2* increased the expression of many genes implicated in aliphatic pathway, including *CYP86A1*, *CYP86B1*, *CYP94A5*, *CYP94C1*, *FARs,* and *KCSs* (**Figure 6**), which covered the synthesis of mainly aliphatic monomers. The positive regulation of AchnMYC2, AchnMYB41 and AchnMYB107 on aliphatic synthesis genes was strongly supported by the accumulation of signature products, ω-hydroxyacids, α, ω-diacids, fatty acids and primary alcohols (**Figure 7**). The long-chain and very long-chain ω-hydroxyacids were mainly produced by CYP86A1 and CYP86B1, respectively, using fatty acids as substrates that were generated by KCS proteins. The CYP86A1, CYP94A5, and CYP94C1 with the function of fatty acid hydroxylase were probably associated with the production of α, ω-diacids (Le Bouquin et al., 2001; Kandel et al., 2007; Höfer et al., 2008). The fatty acids and primary alcohols were mainly generated by KCS and FAR proteins, respectively (Vishwanath et al., 2015). The identity of AchnMYC2, AchnMYB41, and AchnMYB107 directly controlling the transcriptional regulation of *AchnCYP86A1* was performed through yeast one-hybrid and dual-luciferase assays, in which the three TFs could directly interacted with *AchnCYP86A1* promoter to activate the gene expression (**Figure 5**). Collectively, the results suggest that AchnMYC2, AchnMYB41, and AchnMYB107 function as transcriptional activators in regulating *AchnCYP86A1* and probably other aliphatic synthesis genes to coordinate the synthesis of aliphatics.

Suberization is spatially restricted, developmentally regulated and inducible by environmental stimuli. Therefore, suberin synthesis must be highly and strictly regulated at the cell and tissue level (Ranathunge et al., 2011; Verdaguer et al., 2016). Many TFs, including transcriptional activators, and transcriptional repressors, are required to coordinately control suberin synthesis. The negative regulation by transcriptional repressor (such as StNAC103) can occur either in cells where premature deposition of suberin can be detrimental to the proper functioning of the tissue or as a suberin deposition brake in cells where suberin accumulation takes place (Verdaguer et al., 2016). Although *AchnMYB41* and *AchnMYC2* highly expressed in fruit and root, respectively (**Figure 3**), the repressors probably repressed the activation of AchnMYC2 and AchnMYB41 (Fu et al., 2019; Huang et al., 2019), or competed with the two TFs for binding to *AchnCYP86A1* promoter (Zhou et al., 2019), to inhibit the expression of *AchnCYP86A1* in fruit and root (**Figure 3**). However, wounding and ABA treatment probably inhibited the repressors, and induced AchnMYC2, AchnMYB41 and AchnMYB107, to activate *AchnCYP86A1* expression and accumulation of suberin monomers (**Figure 9**).

**Figure 10:**
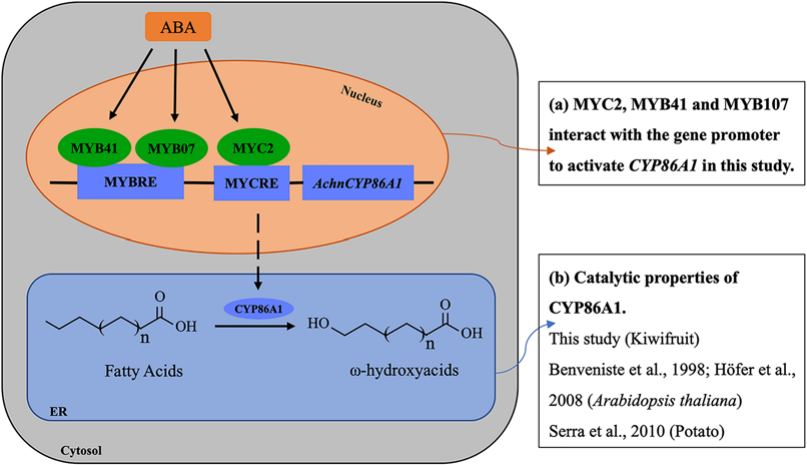
The model of the mechanism of regulating ω-hydroxyacids accumulation through ABA signaling in wound suberization of Kiwifruit. ABA induces AchnMYC2, AchnMYB41 and AchnMYB107 which activate the *AchnCYP86A1* involved in ω-hydroxyacids synthesis. ABA, abscisic acid; MYCRE, MYC recognition element; MYBRE, MYB recognition element.

The mechanisms underlying ABA responses in plants have been intensively studied by biochemical and genetic approaches, which have identified numerous components in the molecular network linking the ABA signal to the stress responses. The components are broadly defined into two large categories, signal transducers and TFs. Among the TFs, a number of MYB and MYC families, such as MYB2, MYB41, MYB96, and MYC2, mediate the ABA signaling in regulation of the stress responsive genes (Abe, 2002; Seo et al., 2009; Agarwal and Jha, 2010; Kosma et al., 2015). In current study, the transcription levels of *AchnCYP86A1* was significantly induced by exogenous ABA (**Figure 9A**), and the gene was individually activated by AchnMYC2, AchnMYB41 and AchnMYB107 (**Figure 5**). Furthermore, *AchnMYC2*, *AchnMYB41,* and *AchnMYB107* were significantly induced by exogenous ABA, but suppressed by FLD (an inhibitor of ABA biosynthesis) (**Figure 9A**). Thus, a model for the involvement of the TFs in the regulation of *AchnCYP86A1* in suberin synthesis *via* the ABA signaling pathway is probably constructed as **Figure 10**. The *AchnMYC2*, *AchnMYB41*, and *AchnMYB107* are induced by ABA, and then *AchnCYP86A1* is activated with the binding of AchnMYC2, AchnMYB41, and AchnMYB107 (**Figure 10A**). Afterward, AchnCYP86A1 catalyzes the ω-hydroxylation of long-chain fatty acids to form the corresponding ω-hydroxyacids (**Figure 10B**).

In conclusion, kiwifruit *AchnCYP86A1* encoding a ω-hydroxylase is involved in the ω-hydroxylation of long-chain fatty acids in suberin synthesis. A regulation mode of AchnMYC2, AchnMYB41 and AchnMYB107 mediated ABA signaling in regulation of *AchnCYP86A1* and probably other aliphatic synthesis genes is presumably elucidated. The AchnMYC2, AchnMYB41 and AchnMYB107 are transcriptional activators in regulation of *AchnCYP86A1* transcript.

## Data Availability Statement

Gene sequence data in this study can be found in the relevant data libraries (Kiwifruit Genome Database, SOL Genomics Network Database, TAIR and NCBI) under gene ID and accession number. 

## Author Contributions

LCM and XPW conceived and designed the experiments. XPW, WJL and XBW performed the experiments. XPW analyzed the data and wrote the manuscript. XYH, WLG, YJY, MZ, CJX and ZSL also contributed to the data interpretation and writing.

## Funding

This work is financially supported by the National Natural Science Foundation of China (31772365, 31972468), the National Key Research and Development Program of China (2018YFD0401303).

## Conflict of Interest

The authors declare that the research was conducted in the absence of any commercial or financial relationships that could be construed as a potential conflict of interest.

## Supplementary Material

The Supplementary Material for this article can be found online at: https://www.frontiersin.org/articles/10.3389/fpls.2019.01650/full#supplementary-material

Figure S1 Phylogenetic analysis of AchnCYP86A1 and AchnMYC2. (a) Phylogenetic tree of AchnCYP86A1 with homologs from other plants. (b) Phylogenetic tree of MYC transcription factors from kiwifruit and Arabidopsis. The phylogenetic tree was constructed with Figtree.

Figure S2 Phylogenetic analysis of MYB transcription factors from kiwifruit and Arabidopsis. The phylogenetic tree was constructed with Figtree.

Figure S3 Relative expression change of glucose metabolism genes in N. benthamiana leaves after 6 days of AchnMYC2, AchnMYB41 and AchnMYB107 overexpression against control (pBI121 empty vector). Error bar represents the standard deviation of three biological replicates. SPS, sucrose-phosphate synthase; HK, hexokinase; AGP, ADP glucose pyrophosphorylase; UGP, UDP glucose pyrophosphorylase.

Figure S4 Yeast one-hybrid assay of Achn031311, Achn173251, Achn313331, Achn318681, Achn136071, Achn310341, Achn313181 and Achn084621 to the AchnCYP86A1 promoter. AD-Rec-p53 with p53-AbAi was used as a positive control, while AD-empty with AchnCYP86A1-AbAi was used as a negative control. Pro, promoter; Ept, empty.

Table S1 Primer sequences were used for quantitative real-time PCR.

Table S2 Primer sequences were used for full-length amplification and vector construction.
