# Supplementary figures and images for: Three Transcription Activators of ABA Signaling Positively Regulate Suberin Monomer Synthesis by Activating Cytochrome P450 CYP86A1 in Kiwifruit
Source: Front Plant Sci. 2020 Jan 10;10:1650. doi: 10.3389/fpls.2019.01650 (PMC6967411; doi:10.3389/fpls.2019.01650)

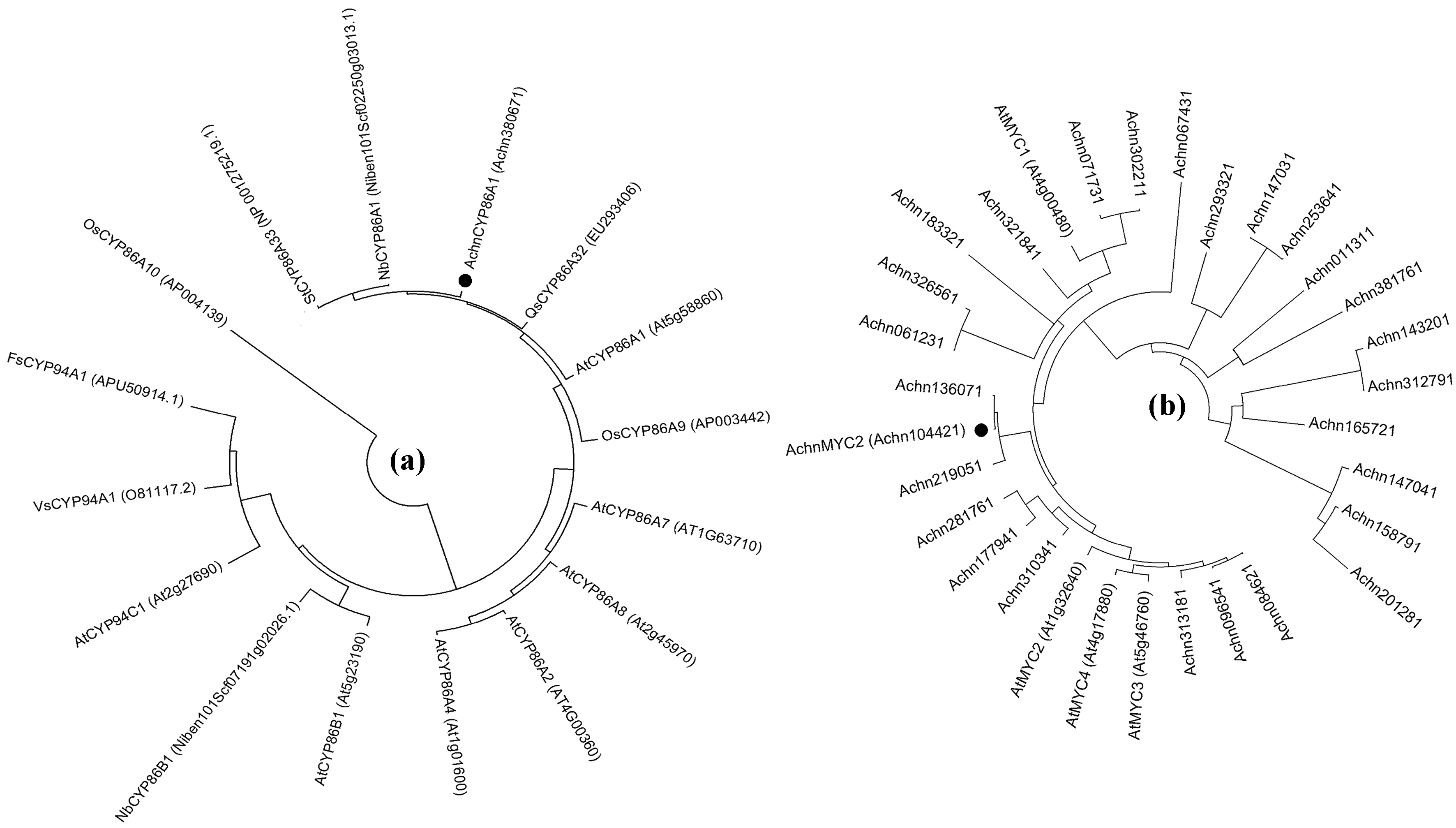

Supplement: Figure S1 — Phylogenetic analysis of AchnCYP86A1 and AchnMYC2. (a) Phylogenetic tree of AchnCYP86A1 with homologs from other plants. (b) Phylogenetic tree of MYC transcription factors from kiwifruit and Arabidopsis. The phylogenetic tree was constructed with Figtree. [file Image_1.jpeg]

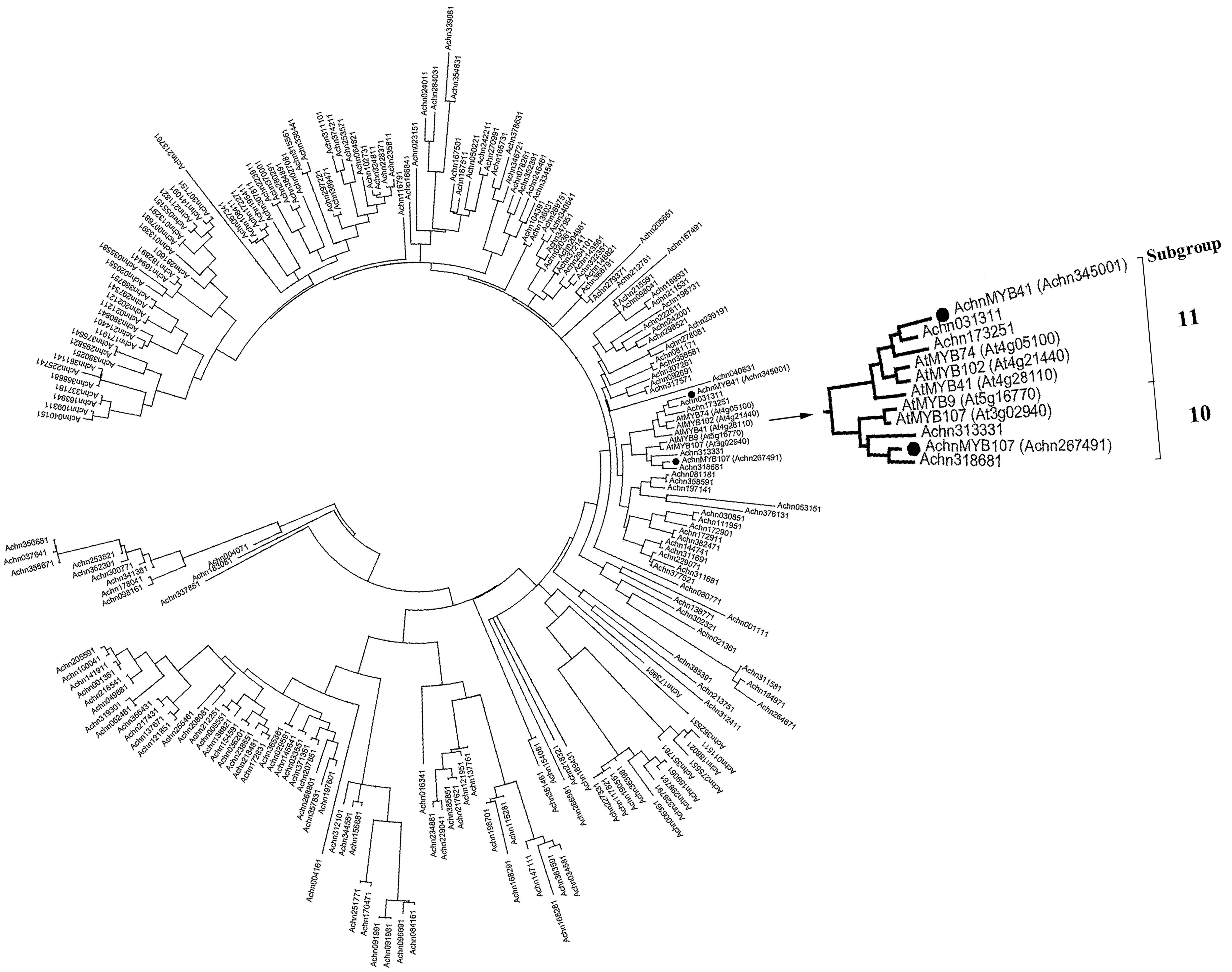

Supplement: Figure S2 — Phylogenetic analysis of MYB transcription factors from kiwifruit and Arabidopsis. The phylogenetic tree was constructed with Figtree. [file Image_2.jpeg]

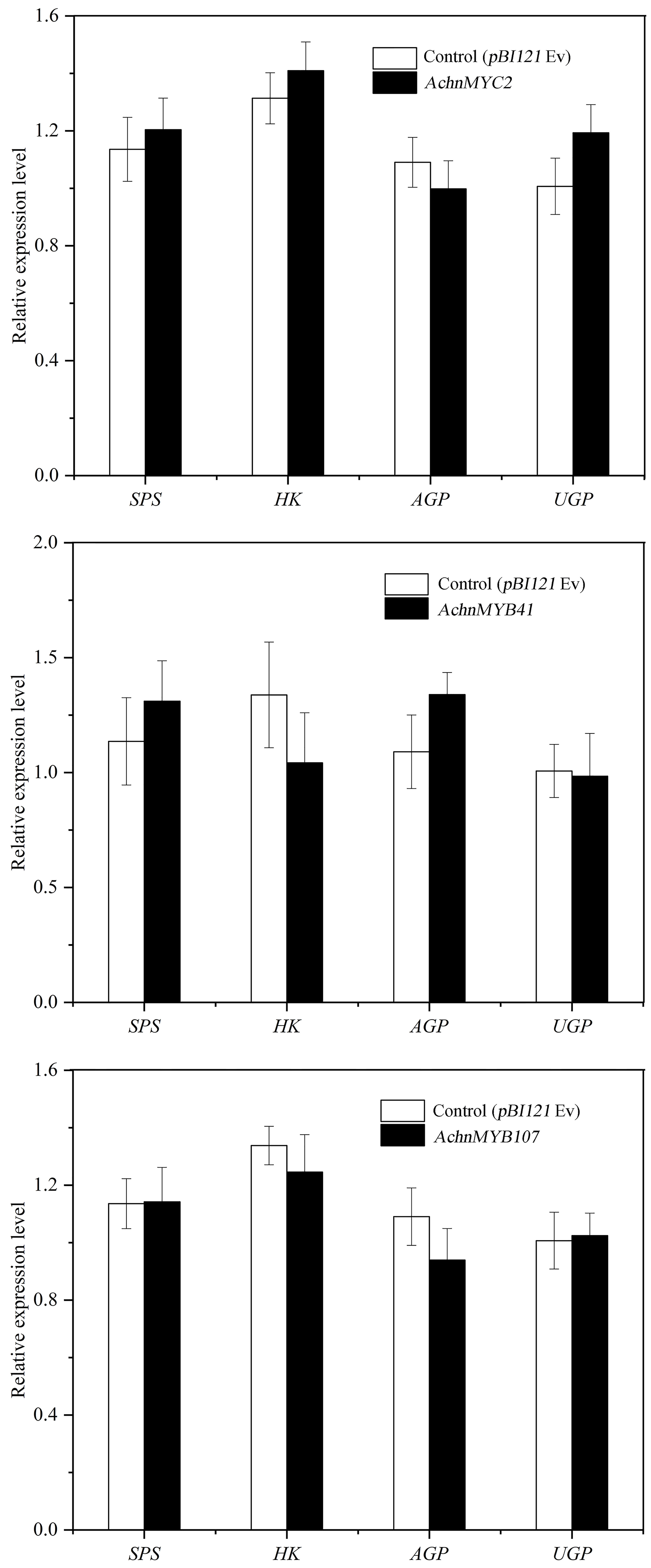

Supplement: Figure S3 — Relative expression change of glucose metabolism genes in N. benthamiana leaves after 6 days of AchnMYC2, AchnMYB41 and AchnMYB107 overexpression against control (pBI121 empty vector). Error bar represents the standard deviation of three biological replicates. SPS, sucrose-phosphate synthase; HK, hexokinase; AGP, ADP glucose pyrophosphorylase; UGP, UDP glucose pyrophosphorylase. [file Image_3.jpeg]

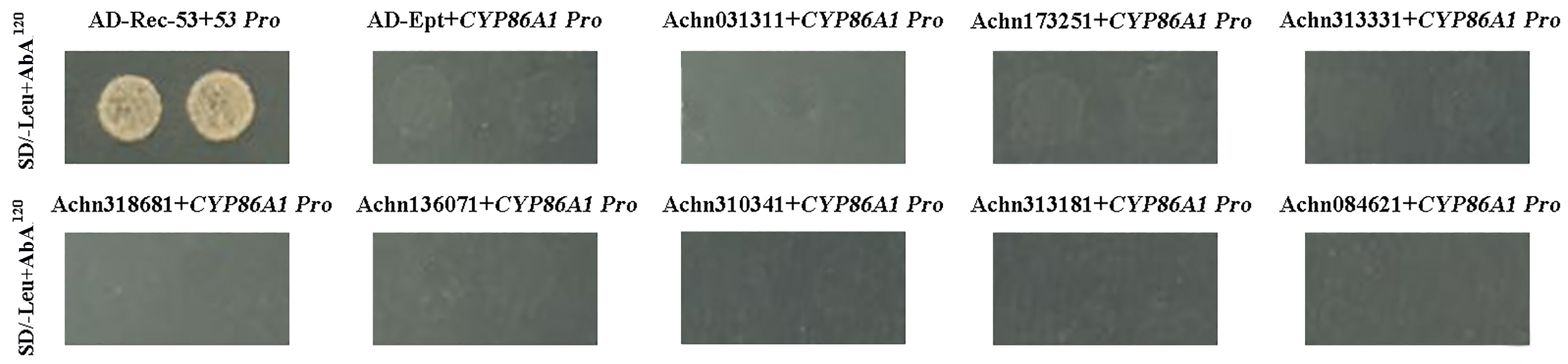

Supplement: Figure S4 — Yeast one-hybrid assay of Achn031311, Achn173251, Achn313331, Achn318681, Achn136071, Achn310341, Achn313181 and Achn084621 to the AchnCYP86A1 promoter. AD-Rec-p53 with p53-AbAi was used as a positive control, while AD-empty with AchnCYP86A1-AbAi was used as a negative control. Pro, promoter; Ept, empty. [file Image_4.jpeg]
